# Suicide among Polish Adolescents—A 20 Year Analysis

**DOI:** 10.3390/ijerph18063190

**Published:** 2021-03-19

**Authors:** Katarzyna Orlewska, Pawel Orlewski, Justyna Klusek

**Affiliations:** 1 Faculty of Health Sciences, Medical University of Warsaw, 02-091 Warsaw, Poland; 2 Institute of Process Engineering, ETH Zurich, 8092 Zurich, Switzerland; porlewski@gmail.com; 3 Department of Surgical Medicine, with the Laboratory of Medical Genetics, Collegium Medicum, Jan Kochanowski University, 25-369 Kielce, Poland; jsklusek@ujk.edu.pl

**Keywords:** suicide, Poland, burden of disease, years of life lost, premature mortality costs, socioeconomic determinants

## Abstract

Background: The study aimed to analyze the burden and territorial diversification of adolescent suicide and the link between suicide attempts and selected socioeconomic variables in Poland. Methods: Rates of suicide by voivodeships for years 1999–2019 were obtained from the General Police Headquarters of Poland database. The burden of premature death was expressed in years of life lost (YLL) and costs of lost productivity, which were estimated using the human capital approach. The link between suicide rates and socioeconomic determinants has been analyzed with Pearson’s correlation coefficient. Results: Over the analyzed period, an increase in suicide attempt rates and a decrease in suicide death rates have been observed. Mean YLL and costs of lost productivity per year amounted to 11,982 and 153,172,415 PLN, respectively. Territorial diversification in suicide attempt rates corresponds to the living condition, poverty, association-based capital, and satisfaction with family situation in individual voivodeships. The number of deaths due to suicide is negatively correlated with an indicator of good living conditions. Conclusions: Our findings provide quantitative evidence of the national impact of suicide and suggest that addressing social capital and poverty may have a role in preventing adolescent mortality due to suicide.

## 1. Introduction

Suicide is the second leading cause of death among adolescents (15–19 years old) worldwide [1], constituting a significant global public health care issue. When family members, friends, and communities of those who attempt or die by suicide are taken into consideration, many millions of people worldwide are affected by suicide every year [2]. Adolescence is a period when one’s personality and identity are shaped. Young people are more likely to lack self-confidence and feel misunderstood, which combined with new challenges they come across may lead to a sense of hopelessness, depression, substance abuse, school failure, which are known risk factors associated with suicidal behavior in this age group [3]. In Poland in years 1999–2006, suicide was the second most common cause of death among children and adolescents, preceded only by traffic accidents [4], and in years 1999–2012, it was the third cause of death among 10–14-year-olds and the second cause of death among 15–19-year-olds [5].

In adolescent and young adults around the world, females present a higher risk of suicide attempt, whereas males are at a higher risk of fatal suicide [6]. Such a tendency is observed among Polish adolescents as well, where boys are more likely to take their own life [7] and girls are more prone to present suicidal thoughts and attempts [8] and are, therefore, four times more likely to be admitted to psychiatric wards [9]. International studies on factors associated with a higher risk of suicide attempts lead to preliminary conclusions that having no close friends, being bullied, interpersonal violence, and previous mental or substance abuse disorder were common risk factors for adolescents of both genders [6,10].

When taking into account each gender separately, dating violence, interpersonal difficulties, and psychiatric disorders (depression, bipolar disorder, eating disorders, posttraumatic stress disorder) were considered as female-specific risk factors, whereas parental separation, family history of suicidal behavior, disruptiveness, and hopelessness were described as risk factors more common among males [6]. According to studies conducted on the Polish adolescent population, girls tend to attempt suicide out of a sense of helplessness, loneliness, rejection, guilt, and due to conflicts with their parents or peers, whereas boys usually die by suicide because of their peers or cyber contacts [11]. Moreover, suicide attempts are more common among girls living in urban areas compared to those living in rural areas [7]. Compared with non-suicidal adolescents, those who present suicidal behavior are more likely to be brought up by single parents, often with a history of alcohol abuse, as well as experience psychological or physical violence from family members [11].

The aims of our study were to examine trends in suicide rates among Polish adolescents aged 13–18 and to evaluate the burden of adolescent suicide on society by analyzing years of life lost (YLL) and costs of premature mortality. We have, furthermore, aimed to assess the territorial diversification in terms of epidemiological indicators and to determine the link between the risk of death due to suicide and suicide attempts and selected socioeconomic variables. Our analysis covered a period of 20 years (from 1999 to 2019). To date, no such comprehensive national studies on this topic have been conducted in Poland.

## 2. Materials and Methods

Anonymous numbers of suicide deaths and attempts among adolescents aged 13–18 were obtained from a database held by the General Police Headquarters of Poland [12]. It is the most reliable source of information, as to date no central database on the number of completed suicides and suicide attempts exists in Poland. The database comprises records from 1999 to 2019, enabling observation over a two-decade period of time. Since 2013, police officers fill in the data directly after determining a suicide at the site, as well as are able to modify the data as a result of investigative proceedings, making the statistics more precise. We have expressed suicide rates as individual numbers in a given year and per 100,000 individuals aged 13–18, according to Polish population tables [13].

YLL were calculated by multiplying the number of deaths due to suicide in the analyzed age group by life expectancy for that age group: YLL = N × SLE, where N is the number of deaths in a given year, and SLE is the standard life expectancy at death. For calculations, the midpoint of age group was considered as age of death. In the base case analysis, SLE was determined using the normative survivorship derived from the most recent World Health Organization Global Health Estimates (WHO GHE) standard life table, which is based on the frontier national life expectancy projected for the year 2050 by the World Population Prospects 2012 and gives a life expectancy of 91.9 years at birth for both sexes [14]. YLL were calculated with a time discount rate at 0% and no age-weighting, as recommended in the Global Burden of Disease 2015 study (GBD 2015) and adopted by the WHO [15,16]. Additionally, YLL were measured applying the up-to-date reference life tables proposed by the Institute for Health Metrics and Evaluation and recently used in the GBD 2015, with a normative standard life expectancy of 86.59 years at birth, not age-weighted nor discounted [15].

Costs of premature mortality were estimated using the human capital approach which measures lost productivity with regard to forgone earnings [17]. For each death, years of potential productive life lost were calculated taken into consideration working lifetime (i.e., from age above 18 till the retirement age, which in Poland is 60 years for women and 65 for men). This work time lost was then valued using sex-specific annual wages for years 1999–2019 [18]. Costs were adjusted for unemployment and labor force participation rates according to labor force characteristics for years 1999–2019 [19,20,21] and discounted at an annual rate of 5% [22]. Based on the analysis of economic activity rate by sex in years 2011–2017 (Statistics Poland 2014, 2018), it was assumed that the economic activity rate for males and females will increase annually by 0.45% and 0.48%, respectively. Future wage growth was estimated at 3.4% based on average country-specific GDP growth from 2000 to 2017. In the base case analysis, costs for each analyzed year have been calculated using economic indicators specific for a given year. In the sensitivity analysis, costs for each analyzed year have been calculated applying economic values for the year 2019. Costs were reported in Polish zloty (PLN) (2020) (1 international dollar = 1.81 PLN in 2020). Adjustment for inflation was performed using local inflation rates [23]. In the second sensitivity analysis, cost of productivity (in current PLN from 1999–2019) was expressed also as multiple of gross domestic product (GDP)/capita at current PLN from 1999–2019.

To determine potential causes of the increase in suicide attempt rates, we analyzed the extent to which the assessments of the territorial diversification in suicide attempt rates correspond with the actual socioeconomic situation in individual regions (voivodeships). We have chosen various social capital and poverty types because they potentially affect health outcomes throughout the life course (Table 1). A selection of variables was determined mainly by the availability of statistical material and requirements governing the choice of determinants. Area-based socioeconomic measurements were retrieved from the Polish Social Cohesion Surveys 2013 and 2015 [24]. The surveys covered the entire Polish population and presented selected measures at a voivodeship level to illustrate territorial diversity in financial situation, experienced poverty, and social relationships. All methodological details have been published elsewhere [24,25]. All 16 voivodeships as units of analysis were chosen because the research question has been formulated at the area-level and the main investigated construct (i.e., socioeconomic determinants) is conceptualized as an area-level attribute. Pearson’s correlation coefficient (PCC) was used to examine the link between the incidence of deaths due to suicide or suicide attempts and socioeconomic determinants. A significance level of *p* < 0.05 was applied (2-tailed tests).

## 3. Results

**Figure 1:**
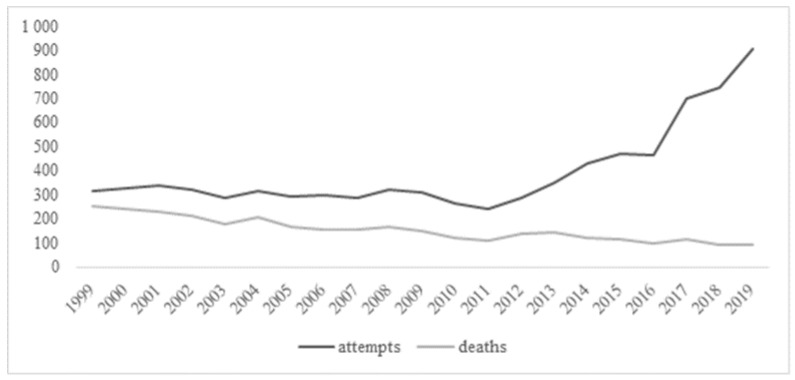
Absolute numbers of reported suicide attempts and deaths due to suicide by year.

In the analyzed age group, an increase in the number of suicide attempts has been observed over a period of 20 years (Figure 1). During recent 5 years, the number has more than doubled—from 428 in 2014 to 905 in 2019. In 1999, 1.3 suicide attempts per 1 suicide death per 100,000 adolescents occurred. Twenty years later, the ratio amounted to a staggering 9.6 (Table 2). The lowest rate of suicide attempts was reported in 2011, amounting to 11.2 per 100,000 adolescents vs. 41.7 per 100,000 adolescents reported in 2019 (Table 2). On the other hand, a declining tendency in the number of deaths due to suicide has been noticed. In 1999, the number of suicides was 251 (11.6 per 100,000 adolescents) and has fallen to 94 (4.3 per 100,000 adolescents) during two decades. A sudden peak in the number of suicides was noted in the years 2004 and 2012 (Figure 1). The lowest number of suicides was observed in 2018 and amounted to 92 (4.24 per 100,000 adolescents) (Table 2). Given the falling number of deaths due to suicide in each analyzed year, a declining tendency has been observed in the YLL values. In 1999, the number of adolescent YLL amounted to a total 19,337 years vs. 7242 years in 2019 (according to WHO GHE) and 16,797 years vs. 6759 years (according to GBD 2015) (Table 2).

### 3.1. Costs of Premature Mortality

Despite a significant decrease in the number of deaths due to suicide, the total cost of lost productivity due to suicide-related premature mortality among adolescents has been reduced by 7% only from 182,286,315 PLN in 1999 to 170,100,369 PLN in 2019 (Table 2). The highest value has been observed in 2008 (208,209,092 PLN), and the mean value amounted to 153,172,415 PLN per year. It is important to stress that during two decades, costs of lost productivity per person have more than doubled—from 726,240 PLN in 1999 to 1,809,578 PLN in 2019, which has been the year with the uppermost registered value. The reasons for such an unexpected upsurge of lost productivity costs per person are changes in economic indicators over the studied period, i.e., growth of economic activity and average gross earnings, as well as a decrease in unemployment rates. Assuming that all years were analyzed according to economic values for the year 2019, the total costs of lost productivity due to adolescent suicide developed in the expected downward direction—from a total of 454,229,172 PLN in 1999 to a total of 170,100,369 PLN in 2019 (Table 2). The same downward trend was seen when costs of lost productivity due to suicide-related premature mortality (in current PLN from 1999–2019) were expressed as multiple of gross domestic product (GDP)/capita at current PLN from 1999–2019.

### 3.2. Territorial Diversity

**Figure 2:**
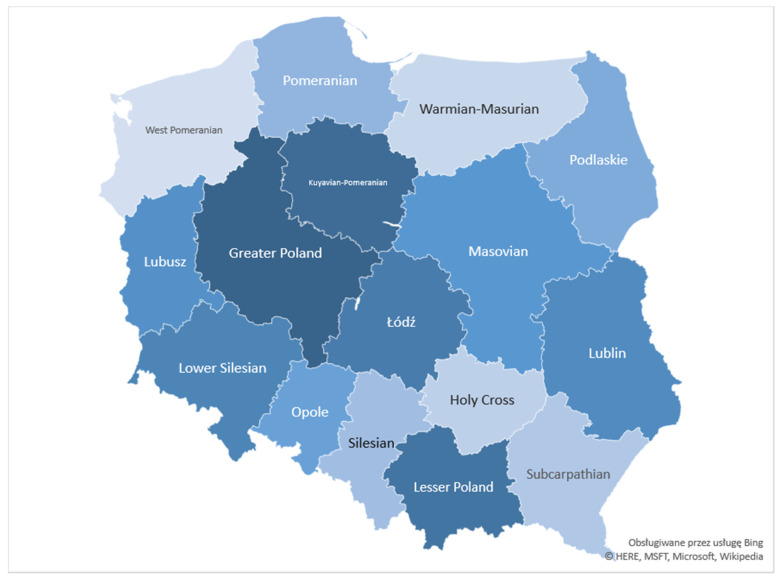
Administrative division of Poland with voivodeship names.

Administrative divisions of Poland with voivodeship names are presented in Figure 2. The analysis of suicide death and attempt rates revealed considerable differences between voivodeships (Supplementary Tables S1 and S2). The voivodeship indicators of diversification of the mortality rate with reference to the average value for Poland (Poland in total = 100%) varied considerably across the analyzed period—in 1999, the values ranged from 47% to 179%, in 2019 from 0.2% to 217%. To compare, the voivodeship indicators of diversification of suicide attempt rates fell within the range from 41% to 209% in 1999 and from 42% to 176% in 2019. Voivodeships with the highest values of suicide death rates in 1999 (Lubusz and Warmian-Masurian) were similarly rated in 2019, while the ranking of voivodeships according to attempt rates have changed—in 1999 West-Pomeranian recorded the highest value of suicide attempts, whereas, in 2019, this voivodeship was rated 12. Since 2014, the highest values of attempts were recorded in Silesian, Warmian-Masurian, and Lodz voivodeships. The rates of deaths related to suicide decreased in all 16 voivodeships, the largest decline (over 80% during the analyzed 20 years) was observed in Holy Cross, West Pomeranian, and Opole voivodeships. In contrast, the rates of attempts displayed a clear upward trend in all except three voivodeships: West-Pomeranian, Greater Poland, and Opole. The highest growth (over 500% during the analyzed 20 years) was seen in Lodz, Pomeranian, Holy Cross, and Lesser Poland voivodeships.

### 3.3. Correlation Analysis

**Figure 3:**
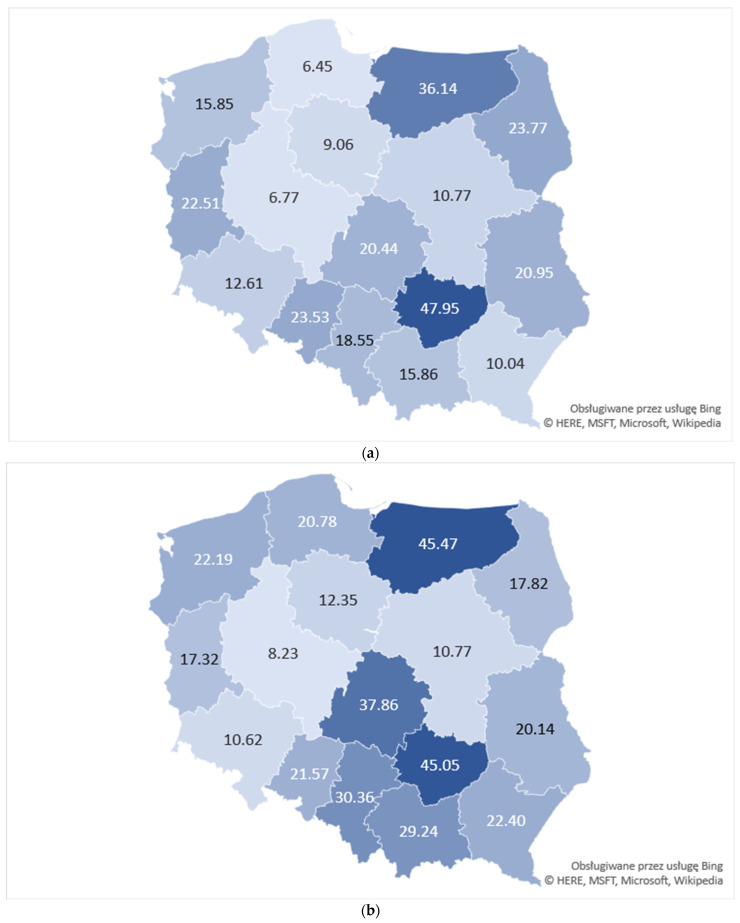
Map of voivodeships presenting suicide attempts per 100,000 adolescents in 2013 (**a**) and 2015 (**b**).

Selected socioeconomic indicators for analyzed years are presented in Supplementary Table S3. Regional diversity of suicide attempt rates in 2013 and 2015 are shown in Figure 3a,b. In 2013, the highest suicide attempt rate (47.95) was recorded in the Holy Cross voivodeship, where at the same time indicators of living condition poverty and association-based capital reached their highest (0.19) and lowest (0.17) values, respectively. Similarly, in 2015, the voivodeship with the highest suicide attempt rate, i.e., Warmian-Mazurian (45.47), was characterized by the highest value of living condition poverty indicator (0.12) and lowest value for association-based capital (0.13).

Pearson’s correlation analysis revealed statistically significant strong positive correlation between suicide attempt rates and living condition poverty (PCC = 0.55; *p* = 0.028 in 2013 and PCC = 0.6; *p* = 0.014 in 2015) and statistically significant strong negative correlation between suicide attempt rates and association-based social capital (PCC = −0.56; *p* = 0.024 in 2015) and satisfaction with family situation (PCC −0.54; *p* = 0.03 in 2013, PCC = −0.5; *p* = 0.05 in 2015). No statistically significant correlation between the suicide attempt rates and income poverty has been observed. Furthermore, in 2013, a statistically significant negative correlation has been recognized between number of deaths due to suicide and indicator of good living conditions (PCC = −0.51; *p* = 0.046).

## 4. Discussion

To date, no such comprehensive analysis of data on Polish adolescent suicide gathered through a period of 20 years and based on official national data has been published. Although several local and national epidemiological studies have been conducted on suicide mortality [1,3,4,5,8,9,11], none of them examines regional diversification of mortality due to suicide and suicide attempts among adolescents, assesses the burden of suicide mortality in terms of years of life lost and costs of lost productivity, nor determines the extent to which the assessments of the territorial diversification in epidemiological indicators correspond with the actual socioeconomic situation in different regions. The findings of our study indicate that adolescent suicide is a great social and economic burden to society, and its prevention is an issue to which priority should be given.

According to our analysis, teenage suicide rates have, on average, declined slightly over the past two decades. It is in line with results of other studies which suggest that suicide among adolescents worldwide has decreased in the past two decades [26,27] with slight increases in recent years [28]. While in 1990, there were, on average, 8.5 suicides per 100,000 teenagers across the Organisation for Economic Co-operation and Development (OECD) countries (aged 15–19), by 2015, this rate had fallen to 7.4. Much of this decline occurred during 2000. Between 1990 and 1999, the OECD average teenage suicide remained fairly stable at around 8.4 suicides per 100,000, but this average fell across the 2000s before reaching a low of 6.3 per 100,000 in 2007 [27]. In 2015, the highest suicide rates—10 or more suicides per 100,000 adolescents aged 15–19—were observed in Canada, Estonia, Latvia, Iceland, and New Zealand. The lowest suicide rates (less than 3 suicides per 100,000 adolescents) were reported in Greece, Israel, Italy, Portugal, and Spain [29]. With an average of 5.25 suicides per 100,000 adolescents in 2015, Poland presents a moderate level of adolescent mortality due to suicide. However, a notable increase in the suicide attempt rates has been observed, especially during the recent years—from 19.7 per 100,000 adolescents in 2014 to 41.7 per 100,000 adolescents in 2019. It should be, furthermore, noticed that according to our study, territorial diversification in suicide attempt rates corresponds with the actual socioeconomic situation in individual voivodeships.

Evidence from the last decade concerning time trends of suicidal thoughts and suicide attempts among adolescents is limited and mainly based on the high school Youth Risk Behavior Survey of the Centers of Disease Control and Prevention of the United States [30,31]. Results of these studies showed that in the US the prevalence of suicidal thoughts in the past 12 months among 14–17-year-olds decreased between 1991 and 2007 (29.0% and 14.5%, respectively) and then increased between 2007 and 2017 (14.5% and 17.2%, respectively). The overall prevalence of having attempted suicide in the past 12 months in 2017 was almost the same as in 1991 (7.1% and 7.3%), with a small significant decrease during this period [30]. In the study performed in the Netherlands, the prevalence of reported suicide attempts during the past 12 months decreased from 2.9% during 2010–2011 to 1.9% during 2014–2015 [32]. A Brazilian study found that there was no trend in the prevalence of suicidal ideation in adolescents from 2006 to 2011, but a significant upward trend was identified in the 2016 survey in comparison to the previous surveys [33]. In general, the comparability of suicide data between countries could be affected by a number of factors, including the time horizon of the analysis and methods of data collection. Studies mentioned above used self-reported data (data were drawn from the Global School-based Student Health Survey (GSHS)), while our study is based on the national database of suicide attempts. According to the experts in the field, the number of suicide attempts is 10–30 times higher compared to completed suicides, in the USA, numbers amount to as high as 100–200 attempts per suicide in adolescents 15–24 years old [34]. The attempts to death ratio reported in our study (9.6 in 2019) are lower, but it is important to stress that we analyzed suicide rates among Polish adolescents aged 13–18, while US data concerned older adolescents aged 15–24. The differences might relate to underreporting or mis-/non-diagnosing, as the majority of those who attempt suicide do not have contact with police or medical services.

No one reason leading to suicide among adolescents has been determined. Inclinations to suicidal behavior depend on individual characteristics and defense mechanisms [9], as well as family bonds, relationships with peers, and social support in general [35]. Family conflicts and school problems were the two most commonly reported causes of youth suicide attempts [7,36]. According to a 2018 survey conducted by the Health Behavior in School-aged Children (HBSC) Polish team, compared to results from a 2014 survey, the level of perceived family and peer support has decreased and a considerable increase in school stress and bullying (especially cyberbullying) has been reported [37]. These may be the reasons for the above-mentioned notable increase in suicide attempts in the last 5 years. Cyberbullying is becoming increasingly dangerous, as today young people are constantly connected and seek acceptance on multiple social platforms, at the same time exposing themselves to online abuse and are thus easily approachable as victims [38]. Besides, easy access to suicidal content on the Internet may lead to the so-called Werther effect, when one suicide entails subsequent suicides [39].

The reported lower level of family and peer support and a corresponding increase of adolescent suicide attempts are in line with our findings, which present statistically significant negative correlations between high suicide attempt rates and satisfaction with a family situation and association-based social capital. As much as 25% of adolescents who attempted suicide admitted trying to seek but failing to receive help from adults [40]. Moreover, self-mutilations, observed mostly among girls as cuts on forearms [7], as well as suicidal ideation [41] have been reported as one of the most important alarming signs of possible suicide attempts in the future. These behaviors may be reduced by the feeling of family and peer support, to which we as a society should pay more attention. In addition, low material status constitutes an increased risk of suicide attempts among adolescents [42]. Our findings of a strong positive correlation between living conditions poverty and suicide attempt further reaffirm this claim. Lack of positive correlation with income poverty implies that low revenue does not determine other poverty forms. Time factor is, therefore, extremely important, as poor living conditions result from unfavorable income situations continuing for a longer period of time.

According to our results, every adolescent’s death due to suicide results in considerable loss of life years and productivity. Over the past two decades, an annual mean of 11,982 YLL and 153,172,415 PLN of lost productivity has been observed. Given the burden of adolescent suicide, during these years, little has been done by the government to prevent premature deaths. Only recently the Ministry of Health developed a National Health Program for years 2016–2020 including, among others, activities aimed at suicide prevention. Efforts undertaken in Poland include improving support for people facing mental problems or living through a suicidal crisis, enhancing the national system of public health surveillance, including collecting data on suicidal behavior and deaths due to suicide, as well as raising media awareness and assuring responsible publications on suicide [43].

As reported by Citizens Network Watchdog Poland [44], there are still only 5.2 child and adolescent psychiatrists per 100,000 juvenile population (the WHO standard is 10). However, adolescent suicide prevention is becoming a recognizable issue of national importance. The National Health Fund has declared a 227% raise in expenditures for child and adolescent psychiatry in 2020, compared to the year 2018 [45]. A new model of child and adolescent psychiatric care is to be enforced on 1 April 2020 [46]. The model is comprised of three reference levels: I—environmental clinics providing psychological and psychotherapeutic care, II—day wards, III—psychiatric hospitals. The main aim is early detection of psychiatric disorders by providing specialistic environmental care clinics operating in the patients nearest environment, which will result in fewer juvenile patients admitted to psychiatric hospitals. Given that depressive disorders are associated with suicidal behavior, the new model can potentially reduce the future number of suicide attempts among adolescents.

On an international scale, the WHO acknowledges the problem of suicide and encourages countries to develop or strengthen comprehensive suicide prevention strategies. In 2014, a first-ever global suicide prevention report has been published, calling for action to employ national suicide prevention strategies which should include key elements brought together in the LIVE LIFE approach [47]. Leadership, Interventions, Vision, and Evaluation are the pillars of core interventions, i.e., Less means (limiting access to means of suicide), Interaction with media, Formation of life skills in the young, and Early identification, management, and follow-up. This approach may result in an even more considerable reduction in adolescent suicide rates when combined with existing national preventive programs.

Our study strongly relies on the registry of deaths run by the General Police Headquarters of Poland. The accuracy of such databases may be influenced by misclassifying the primary cause of death. Suicide remains a sensitive topic and the rates may be underestimated due to stigma and poor surveillance systems. To date, two national databases on suicide are available in Poland—one is held by the General Police Headquarters of Poland, the second by the Central Statistics Office. The authors decided to base the calculations on police rather than Central Statistics Office data, as it covers information not only on deaths due to suicide but also on suicide attempts. Despite its limitations, the General Police Headquarters database is currently the most reliable and most commonly employed source of data on suicide deaths and attempts in Poland. In April 2020, the working team for the prevention of suicide and depression at the Public Health Council of the Ministry of Health adopted a resolution on the implementation of work on the National Database for Monitoring and Prevention of Suicidal Behavior. Such a registry, if introduced, might be useful in further studies.

The major limitation of our study is the conservativeness of the prevalence data, as possibly a considerate amount of suicide attempts and deaths remain underestimated in the national databases. Data availability bias might exist due to the varying completeness of police reports. It is usually found that many more attempts occur than are ever reported to police or medical personnel [48].

Some studies demonstrate that the suicide classification rates are underestimated by approximately 30–40% [49,50]. Furthermore, no individual socioeconomic characteristics of adolescents who attempted or died due to suicide are available. Socioeconomic variables were measured on voivodeship level and disparities documented in our study do not reflect the experience of an individual. The multifactorial nature of suicide indicates that it is caused by a combination of environmental, lifestyle, and socioeconomic factors developing over one’s lifetime. It, therefore, seems reasonable to analyze suicide risk in an environmental and socioeconomic context.

Association between the risk of suicide attempts and socioeconomic variables was examined for years 2013 and 2015 because only for these years a complete set of data on various poverty and social capital types was available. However, analyzed variables depend not only on the present but on the past as well. Living conditions poverty and social capital are strongly related to the current and previous situation. Thus, even measured at a given point of time, such calculations may provide data about lifetime exposure to these variables in individual regions.

## 5. Conclusions

Our analysis has filled the information gap in knowledge of adolescent suicide incidence, burden, and regional diversity in Poland. The trend analysis of suicide mortality and attempt rates adds insight into variables over a period of time and helps to identify potential planning issues to achieve the goal of reducing the rates of adolescent suicide. The findings of our study suggest that addressing living conditions poverty and social capital may play an important role in preventing suicide among Polish adolescents. The authors hope that this paper will further contribute to understanding the burden of adolescent suicide not only on a national but on a global level as well.

## Figures and Tables

**Table 1:** Definitions of socioeconomic determinants used in the study (according to the Polish Social Cohesion Survey).

Thematic Area	Indicator	Definition
Social capital	Association-based social capital	% of persons aged 16 or more who declared their involvement in at least one organisation, association, or formal group
	Satisfaction with family situation	% of persons aged 16 years or more, declaring that they were satisfied with their current family situation
Poverty	Income poverty	% of households in which the monthly equivalised income at household’s disposal (within 12 months preceding the survey) was lower than the value regarded as the poverty threshold. The poverty threshold was assumed at 60% of the median equivalised income, i.e., income comparable between households with different demographic structures
	Living conditions poverty	% of households in which at least 10 indications of poor living conditions were observed, based on the list of 30 symptoms concerning the dwelling quality, the provision of durable consumer goods, and the deprivation of various types of consumer needs

**Table 2:** Suicide attempt and death rates; suicide attempt to death ratio; YLL per all deaths due to suicide according to WHO GHE and GBD 2015; and costs of lost productivity per all deaths due to suicide (base case and sensitivity analysis). YLL—years of life lost, WHO GHE—World Health Organization Global Health Estimates, GBD—Global Burden of Disease.

	Attempts /100,000	Deaths /100,000	Attempts /death /100,000	YLL/All Deaths acc. WHO GHE	YLL/All Deaths acc. GBD 2015	Costs of Lost Productivity/All Deaths (in PLN, 2020)		Cost of Lost Productivity /GDP Per Capita
						Base Case Analysis	Sensitivity Analysis	
1999	14.6	11.6	1.3	19,337	16,797	182,286,315.30	454,223,172.10	5873
2000	15.2	11.1	1.4	18,490	16,061	176,782,502.60	434,316,977.30	5624
2001	15.5	10.5	1.5	17,642	15,325	159,399,636.40	414,410,782.50	5266
2002	14.7	9.8	1.5	16,332	14,187	143,655,545.60	383,646,663.30	4728
2003	13.2	8.3	1.6	13,867	12,046	124,224,452.10	325,737,733.00	3946
2004	14.6	9.6	1.5	16,024	13,919	196,665,099.60	376,408,047.00	5759
2005	13.5	7.7	1.8	12,866	11,176	161,616,281.10	302,212,230.10	4644
2006	13.7	7.2	1.9	12,018	10,440	160,681,561.00	282,306,035.30	4311
2007	13.3	7	1.9	11,787	10,239	181,540,059.20	276,877,073.00	4446
2008	14.8	7.6	1.9	12,789	11,109	208,209,092.20	300,402,576.00	4893
2009	14.4	7	2.1	11,710	10,172	195,305,946.80	275,067,419.00	4479
2010	12.3	5.5	2.2	9168	7963	150,594,102.40	215,348,834.60	3400
2011	11.2	5.1	2.2	8551	7428	117,833,706.10	200,871,602.00	2537
2012	13.2	6.4	2.1	10632	9235	151,882,950.50	249,732,262.00	3298
2013	16	6.6	2.4	11094	9636	163,673,765.90	260,590,186.40	3583
2014	19.7	5.7	3.5	9553	8298	145,230,212.10	224,397,105.00	3080
2015	21.6	5.2	4.1	8783	7629	145,218,552.30	206,300,564.20	2898
2016	21.5	4.7	4.6	7781	6759	134,398,252.20	182,775,061.30	2573
2017	32.3	5.3	6.1	8860	7696	186,893,350.60	208,110,218.30	3381
2018	34.4	4.2	8.1	7088	6157	15,465,630.40	166,488,174.60	2681
2019	41.7	4.3	9.6	7242	6290	170,100,368.60	170,107,482.80	2765
mean	18	7	3	11,982	10,408	153,172,414.54	281,444,295.23	4208

## Data Availability

All data are publicly available.

## Supplementary Materials

The following are available online https://www.mdpi.com/1660-4601/18/6/3190/s1, Table S1. The number of suicide deaths per 100,000 adolescents for analysed years according to voivodeships; Table S2. The number of suicide attempts per 100,000 adolescents for analysed years according to voivodeships; Table S3. Selected socioeconomic indicators for analysed years.

## References

[1] Shain B. (2016). Suicide and suicide attempts in adolescents. Pediatrics.

[2] Cerel J., Brown M., Maple M., Singleton M., van de Venne J., Moore M., Van de Venne J., Moore M., Flaherty C. (2018). How Many People Are Exposed to Suicide? Not Six. Suicide Life-Threat. Behav..

[3] Picazo-Zappino P.Z. (2014). Suicide among children and adolescents: A review. Actas Esp. Psiquiatr..

[4] Kułaga Z. (2009). Aktualne trendy zewnętrznych przyczyn zgonów dzieci i młodzieży w Polsce. Probl. Hig. Epidemiol..

[5] Grajda A., Kułaga Z., Gurzkowska B., Góźdź M., Wojtyło M., Litwin M. (2017). Trends in external causes of child and adolescent mortality in Poland, 1999–2012. Int. J. Public Health.

[6] Miranda-Mendizabal A., Castellví P., Parés-Badell O., Alayo I., Almenara J., Alonso I., Almenara J., Alonso I., Blasco M.J., Cebria A. (2019). Gender differences in suicidal behavior in adolescents and young adults: Systematic review and meta-analysis of longitudinal studies. Int. J. Public Health.

[7] Mroczkowska-Juchkiewicz A., Krawiec P., Pawłowska-Kamieniak A., Gołyska D., Kominek K., Pac-Kożuchowska E. (2016). Intentional spoisonings in urban and rural children—A 6-year retrospective single centre study. Ann Agric. Environ. Med..

[8] Makara-Studzinska M., Sygit K., Sygit M., Gozdziewska M., Zubilewicz J., Krys-Noszczyk K. (2012). Analysis of the phenomenon of attempted suicides in 1978–2010 in Poland, with particular emphasis on rural areas of lublin province. Ann. Agric. Environ. Med..

[9] Gmitrowicz A., Wolanek U., Madej A., Studzińska M.-M. (2015). Motywy podejmowania prób samobójczych przez młodzież w wieku 13–19 lat. J. Educ. Heal Sport..

[10] Campisi S.C., Carducci B., Akseer N., Zasowski C., Szatmari P., Bhutta Z.A. (2020). Suicidal behaviours among adolescents from 90 countries: A pooled analysis of the global school-based student health survey. BMC Public Health.

[11] Zygo M., Pawłowska B., Potembska E., Dreher P., Kapka-Skrzypczak L. (2019). Prevalence and selected risk factors of suicidal ideation, suicidal tendenciesand suicide attempts in young people aged 13–19 years. Ann. Agric. Environ. Med..

[12] General Police Headquarters Suicide and Suicide Attempts Rates in Poland. http://statystyka.policja.pl/.

[13] Statistics Poland Polish Population Tables. https://stat.gov.pl/.

[14] World Health Organization WHO Methods and Data Sources for Global Burdes and Information Systems WHOn of dIsease Estimates 2000–2011. Glob Heal Estim Tech Pap WHO/HIS/HSI/GHE/20134. http://www.who.int/healthinfo/statistics/GlobalDALYmethods_2000_2011.pdf?ua=1.

[15] Wang H., Naghavi M., Allen C., Barber R.M., Carter A., Casey D.C., Charlson F.J., Chen A.Z., Coates M.M., Coggeshall M. (2016). Global, regional, and national life expectancy, all-cause mortality, and cause-specific mortality for 249 causes of death, 1980–2015: A systematic analysis for the Global Burden of Disease Study 2015. Lancet.

[16] Zhou M., Wang H., Zeng X., Yin P., Zhu J., Chen W., Li X., Wang L., Wang L., Liu Y. (2015). Global, regional, and national age-sex specific all-cause and cause-specific mortality for 240 causes of death, 1990–2013: A systematic analysis for the Global Burden of Disease Study 2013. Lancet.

[17] Pike J., Grosse S.D. (2018). Friction Cost Estimates of Productivity Costs in Cost-of-Illness Studies in Comparison with Human Capital Estimates: A Review. Appl. Health Econ. Health Policy.

[18] Social Insurance Institution (ZUS) Average Salary for Years 1950–2019. https://www.zus.pl/baza-wiedzy/skladki-wskazniki-odsetki/wskazniki/przecietne-wynagrodzenie-w-latach.

[19] Statistics Poland (2004). Women and Men on the Labour Market 2004.

[20] Statistics Poland (2014). Women and Men on the Labour Market 2014.

[21] Statistics Poland (2018). Women and Men on the Labour Market 2018.

[22] Agency for Health Technology Assessment and Tariff System (2016). HTA Guidelines.

[23] Historic Inflation Poland (CPI). https://www.inflation.eu/en/inflation-rates/poland/historic-inflation/cpi-inflation-poland.aspx.

[24] Bieńkuńska A., Ciecieląg P., Haponiuk M., Nałęcz S., Sobestjański K., Wieczorkowski R., Jachowicz I., Kolasa E., Piasecki T., Szkopiecka K. (2017). Regional Diversity of Quality of Life in 2015. https://stat.gov.pl/obszary-tematyczne/warunki-zycia/dochody-wydatki-i-warunki-zycia-ludnosci/terytorialne-zroznicowanie-jakosci-zycia-w-polsce-w-2015-r-,25,1.html.

[25] Verger D., Lebrere A., Bienkunska A., Ciecieląg P., Nałęcz S., Piaskowski P., Wieczorkowski R., Lednicki B. (2014). Quality of life. Social capital, poverty and social exclusion in Poland. http://stat.gov.pl/en/topics/living-conditions/living-conditions/quality-of-life-social-capital-poverty-and-social-exclusion-in-poland,4,1.html.

[26] McLoughlin A.B., Gould M.S., Malone K.M. (2015). Global trends in teenage suicide: 2003–2014, QJM. Int. J. Med..

[27] CO4.4: Teenage Suicides (15–19 Years Old). OECD Family Database. http://www.oecd.org/els/family/database.html.

[28] Belsher B.E., Smolenski D.J., Pruitt L.D., Bush N.E., Beech E.H., Workman D.E., Morgan R.L., Evatt D.P., Tucker J., Skopp N.A. (2019). Prediction Models for Suicide Attempts and Deaths: A Systematic Review and Simulation. JAMA Psychiatry.

[29] (2009). Social Affairs. CO4. 1: Teenage Suicide (15–19 years old) Definitions and methodology Key Findings. www.oecd.org/els/family/database.html.

[30] Kann L., McManus T., Haris W.A. (2018). Youth risk behavior surveillance United States, 2017. MMWR Surveill. Summ..

[31] Lindsey M.A., Sheftall A.H., Xiao Y., Joe S. (2019). Trends of suicidal behaviors among high school students in the United States: 1991–2017. Pediatrics.

[32] Van Vuuren C.L., van der Wal M.F., Cuijpers P., Chinapaw M.J.M. (2020). Sociodemographic Differences in Time Trends of Suicidal Thoughts and Suicide Attempts Among Adolescents Living in Amsterdam, The Netherlands. Crisis.

[33] Soares F.C., Hardman C.M., Rangel Junior J.F.B., Bezerra J., Petribú K., Mota J., de Barros M.V.G., Lima R.A. (2020). Secular trends in suicidal ideation and associated factors among adolescents. Braz. J. Psychiatry..

[34] Bachmann S. (2018). Epidemiology of Suicide and the Psychiatric Perspective. Int. J. Environ. Res. Public Health.

[35] Stradomska M., Wolińska J., Marczak M. (2016). Uwarunkowania prób samobójczych u nastoletnich pacjentów szpitali i klinik psychiatrycznych w perspektywie psychologicznej. Psychiatr Psychol. Klin..

[36] Bąbik Aleksandra O.D. (2014). Uwarunkowania i profilaktyka samobójstw wśród dzieci i młodzieży w Polsce. Dziecko KrzywdzoneTeoria Bad. Prakt..

[37] Mazur J., Małkowska-Szkutnik A. (2018). Zdrowie Uczniów w 2018 Roku Na Tle Nowego Modelu Badań HBSC. Warsaw.

[38] Pyżalski J. (2012). Agresja Elektroniczna i Cyberbullying Jako Nowe Ryzykowne Zachowania Młodzieży. http://repozytorium.amu.edu.pl/handle/10593/5939.

[39] Kropiwnicki P., Gmitrowicz A. (2013). Światowe Inicjatywy W Zakresie Profilaktyki Samobójstw. Psychiatr. Psychol. Klin..

[40] Szymańska J. (2016). Zapobieganie Samobójstwom Dzieci i Młodzieży.

[41] Farrell C.T., Moledina Z., Katta M. (2019). Suicidal thoughts in low-income adolescents: A longitudinal analysis. Int. J. Public Health.

[42] Koszewska I., Średniawa M., Koszewska J. (2014). Internet i Nowe Technologie w Zapobieganiu Samob Ójstwom Raport na Zlecenie Maic.

[43] Gmitrowicz A., Baran A., Ostaszewski K. (2019). The Milestones of Development of National Suicide Preventive Strategy in Poland.

[44] Maślankiewicz R., Bójko M. (2019). Psychiatria Dzieci i Młodzieży w Polsce.

[45] Polish Press Agency (2020). Interview with the Deputy Minister of Health. http://nauka.pap.pl/palio/html.run?_Instance=cms_nauka.pap.pl&_PageID=7&dep=338020&_CheckSum=1843758126.

[46] National Health Fund (2020). Decree Nr 17/2020/DEF. https://www.nfz.gov.pl/zarzadzenia-prezesa/zarzadzenia-prezesa-nfz/zarzadzenie-nr-172020def,7125.html.

[47] World Health Organization (2014). Preventing Suicide.

[48] Katz C., Bolton J., Sareen J. (2016). The prevalence rates of suicide are likely underestimated worldwide: Why it matters. Soc. Psychiatry Psychiatr. Epidemiol..

[49] Auger N., Burrows S., Gamache P., Hamel D. (2016). Suicide in Canada: Impact on injuries of undetermined intent on regional rankings. Inj. Prev..

[50] Bakst S.S., Braun T., Zucker I., Amitai Z., Shohat T. (2016). The accuracy of suicide statistics: Are true suicide deaths misclassified?. Soc. Psychiatry Psychiatr. Epidemiol..

