# Supplementary material for: Suicide among Polish Adolescents—A 20 Year Analysis
Source: Int J Environ Res Public Health. 2021 Mar 19;18(6):3190. doi: 10.3390/ijerph18063190 (PMC8003452; doi:10.3390/ijerph18063190)
Supplement: Supplementary file 1 [file ijerph-18-03190-s001.pdf]

Supplementary Table S1. The number of suicide deaths per 100,000 adolescents for analysed years according to voivodeships.

	Kuyavian-Pomeranian	Podlaskie	Pomeranian	Lubusz	Silesian	Holy Cross	Lesser Poland	Lodz	Lublin	Warmian-Masurian	Opole	Greater Poland	Subcarpathian	West Pomeranian	Lower Silesian	Masovian
1999	13.18	17.82	5.73	20.78	12.23	10.17	5.45	10.6	11.28	18.65	17.65	16.94	10.04	16.91	7.3	9.14
2000	7.41	23.77	10.75	10.39	9.7	23.25	9.41	11.36	10.47	8.16	21.57	7.74	13.9	10.57	14.61	7.83
2001	4.94	19.31	8.6	24.24	7.59	15.98	7.43	12.11	16.92	12.82	11.76	12.1	10.04	6.34	13.28	7.18
2002	5.76	16.34	9.31	12.12	9.7	13.08	9.91	8.33	12.89	11.66	19.61	9.19	7.72	9.51	11.29	6.53
2003	5.76	14.85	5.73	15.58	8.01	5.81	7.43	8.33	14.5	10.49	5.88	8.23	9.27	5.28	11.95	4.9
2004	18.12	5.94	9.31	22.51	7.59	5.81	8.42	7.57	16.11	10.49	17.65	5.81	3.09	13.74	13.28	6.53
2005	11.53	10.4	9.31	12.12	7.59	2.91	9.41	2.27	9.67	13.99	3.92	5.81	7.72	9.51	7.3	5.22
2006	10.71	8.91	8.6	13.85	5.9	14.53	6.94	1.51	12.09	9.33	3.92	5.32	10.04	2.11	6.64	5.22
2007	8.23	17.82	8.6	6.93	6.75	7.27	3.47	6.06	10.47	9.33	3.92	5.81	7.72	7.4	7.3	5.22
2008	4.94	20.8	5.73	10.39	4.22	10.17	6.94	9.84	16.92	6.99	13.73	4.84	8.5	3.17	5.98	6.86
2009	4.94	13.37	2.87	10.39	7.17	5.81	3.47	14.39	9.67	5.83	7.84	7.74	6.95	2.11	2.66	9.14
2010	7.41	8.91	4.3	8.66	2.95	7.27	3.96	4.54	8.06	9.33	7.84	3.39	3.09	7.4	5.31	6.2
2011	3.29	5.94	7.17	10.39	5.06	4.36	3.47	4.54	4.83	10.49	5.88	2.9	4.63	8.45	2.66	5.55
2012	11.53	11.88	5.73	10.39	6.32	7.27	2.48	6.81	6.45	13.99	1.96	3.39	6.18	3.17	4.65	7.18
2013	5.76	8.91	3.58	8.66	7.59	2.91	4.46	7.57	9.67	9.33	11.76	2.9	6.18	8.45	9.96	6.2
2014	2.47	7.43	4.3	3.46	3.37	5.81	2.97	7.57	8.06	5.83	5.88	4.35	6.95	9.51	13.28	4.9
2015	8.23	2.97	5.02	6.93	2.11	2.91	2.97	12.87	8.06	5.83	5.88	2.9	6.95	6.34	3.98	5.22
2016	3.29	2.97	7.88	8.66	2.11	4.36	1.98	6.06	8.86	10.49	5.88	3.87	2.32	5.28	5.98	3.59
2017	4.12	8.91	4.3	10.39	5.06	1.45	4.96	6.06	4.03	9.33	0	3.87	3.09	3.17	7.97	6.86
2018	6.59	0	5.02	5.19	2.95	1.45	2.97	3.79	7.25	3.5	5.88	2.42	3.09	9.51	4.65	4.9
2019	4.12	2.97	3.58	6.93	3.37	0	4.46	6.81	4.83	9.33	1.96	4.84	4.63	2.11	5.98	3.26
mean	7.25	10.96	6.45	11.38	6.06	7.27	5.38	7.57	10.05	9.77	8.59	5.92	6.77	7.14	7.91	6.08

Supplementary Table S2. The number of suicide attempts per 100,000 adolescents for analysed years according to voivodeships.

	Kuyavian-Pomeranian	Podlaskie	Pomeranian	Lubusz	Silesian	Holy Cross	Lesser Poland	Lodz	Lublin	Warmian-Masurian	Opole	Greater Poland	Subcarpathian	West Pomeranian	Lower Silesian	Masovian
1999	14.82	28.22	7.17	22.51	15.18	10.17	5.95	11.36	13.7	20.98	25.49	21.77	13.13	30.64	11.95	10.12
2000	8.23	29.71	15.05	12.12	13.91	23.25	14.87	12.11	16.11	9.33	29.41	11.61	16.99	31.7	20.58	8.81
2001	8.23	28.22	13.61	32.9	13.91	18.89	11.4	19.69	23.36	15.16	15.69	17.9	10.81	24.3	18.59	7.51
2002	5.76	23.77	10.75	27.71	15.18	15.98	15.86	15.14	16.92	17.49	27.45	19.35	11.59	23.24	11.95	7.18
2003	8.23	19.31	8.6	29.44	19.4	7.27	9.91	9.84	19.34	16.32	19.61	13.55	11.59	22.19	13.28	6.2
2004	23.06	14.85	10.03	27.71	13.07	8.72	13.38	10.6	23.36	12.82	27.45	13.06	9.27	28.53	15.27	9.47
2005	16.47	20.8	15.76	24.24	13.91	2.91	11.89	6.06	14.5	30.31	23.53	8.71	15.45	23.24	8.63	9.14
2006	12.35	14.85	20.06	20.78	13.91	17.44	11.89	7.57	20.14	24.48	25.49	9.68	16.99	16.91	9.29	7.18
2007	11.53	25.25	13.61	15.58	13.07	13.08	9.91	15.14	16.11	31.48	15.69	8.23	12.36	20.08	14.61	6.86
2008	12.35	31.19	8.6	24.24	12.65	11.62	9.91	12.87	33.84	32.64	23.53	10.65	16.99	19.02	9.29	8.49
2009	9.06	23.77	14.33	24.24	16.44	10.17	8.42	22.72	17.73	26.81	25.49	9.68	14.68	8.45	7.97	13.71
2010	11.53	14.85	12.18	24.24	10.54	10.17	9.41	15.9	10.47	26.81	41.18	4.84	7.72	24.3	5.98	10.12
2011	9.06	19.31	9.31	24.24	14.76	4.36	6.44	10.6	11.28	34.97	13.73	7.74	6.18	22.19	4.65	7.83
2012	14	20.8	8.6	15.58	14.34	26.16	8.92	18.17	14.5	34.97	25.49	4.35	7.72	11.62	6.64	12.73
2013	9.06	23.77	6.45	22.51	18.55	47.95	15.86	20.44	20.95	36.14	23.53	6.77	10.04	15.85	12.61	10.77
2014	4.94	28.22	15.05	19.05	21.93	50.86	22.3	34.83	24.98	32.64	17.65	10.16	11.59	24.3	17.26	13.06
2015	12.35	17.82	20.78	17.32	30.36	45.05	29.24	37.86	20.14	45.47	21.57	8.23	22.4	22.19	10.62	10.77
2016	8.23	23.77	22.21	17.32	34.58	42.14	25.27	38.62	16.11	44.3	23.53	11.61	15.45	17.96	17.26	9.47
2017	14.82	43.08	37.98	22.51	52.71	49.4	36.67	63.6	20.95	45.47	17.65	15.97	26.26	23.24	31.2	20.24
2018	18.12	38.62	41.56	24.24	49.33	61.03	38.16	62.09	33.84	40.8	27.45	20.32	33.99	24.3	30.54	20.24
2019	17.29	68.33	48.72	39.83	57.77	65.39	47.57	73.45	46.73	46.63	17.65	20.32	37.85	31.7	49.79	22.52
mean	11.88	26.60	17.16	23.25	22.17	25.81	17.30	24.70	20.72	29.81	23.25	12.12	15.67	22.19	15.62	11.07

Supplementary Table S3. Selected socioeconomic indicators for analysed years.

Voivodeship	Income poverty		Living conditions poverty		Association-based social capital		Satisfaction with family situation	
	2013	2015	2013	2015	2013	2015	2013	2015
Kuyavian-Pomeranian	17%	17%	14%	8%	23%	20%	79%	79%
Podlaskie	20%	17%	9%	5%	29%	31%	68%	74%
Pomeranian	14%	13%	12%	9%	23%	16%	78%	79%
Lubusz	15%	11%	15%	10%	24%	21%	78%	80%
Silesian	12%	9%	11%	7%	21%	19%	78%	81%
Holy Cross	20%	24%	19%	11%	17%	16%	77%	72%
Lesser Poland	18%	15%	12%	8%	25%	20%	75%	79%
Lodz	13%	15%	17%	11%	22%	14%	71%	73%
Lublin	23%	27%	16%	10%	38%	24%	70%	73%
Warmian-Masurian	15%	17%	20%	12%	19%	13%	67%	74%
Opole	17%	11%	10%	8%	31%	25%	82%	79%
Greater Poland	13%	13%	9%	6%	21%	20%	80%	81%
Subcarpathian	20%	21%	13%	8%	27%	21%	73%	75%
West Pomeranian	19%	16%	18%	12%	21%	19%	67%	74%
Lower Silesian	11%	11%	14%	8%	22%	19%	75%	78%
Masovian	12%	11%	14%	8%	22%	22%	72%	76%
Mean	16%	16%	14%	9%	24%	20%	74%	77%
